# A preliminary analysis of the diet composition of overwintering Bean geese (*Anser fabalis*) and greater white-fronted geese (*A. albifrons*) in Korea using PCR on fecal samples

**DOI:** 10.1080/19768354.2017.1308437

**Published:** 2017-04-13

**Authors:** Min Kyung Kim, Sang-im Lee, Baek-Jun Kim, Sang Don Lee

**Affiliations:** a Department of Environmental Science and Engineering, College of Engineering, Ewha Womans University, Seoul, Korea; b School of Undergraduate Studies, Daegu-Gyeongbuk Institute of Science and Technology, Daegu, Korea; c National Institute of Ecology, Seocheon, South Korea

**Keywords:** Diet, feces, *Anser fabalis*, *Anser albifrons*, South Korea

## Abstract

Bean geese (*Anser fabalis*) and Greater white-fronted geese (*Anser albifrons*) are the dominant wintering waterfowl in South Korea. Although they are commonly observed in estuaries and rice fields during the winter, the diet composition of the geese during the winter has rarely been studied. In this study, we provide the results from preliminary analyses on the diet of these two geese species overwintering in Daebu Island of South Korea. We used a total of 13 fecal samples from Bean geese (*n* = 4) and Greater white-fronted geese (*n* = 9), and performed a BLAST search for the sequences obtained from 87 clones (*n* = 36 for Bean geese and *n* = 51 for Greater white-fronted geese). The diet of Bean geese consisted of five families of plants: Caryophyllaceae (75.0%), Poaceae (13.9%), Asteraceae (5.5%), Polygonaceae (2.8%) and Cucurbitacea (2.8%). On the other hand, the diet of Greater white-fronted geese consisted of 6 families of plants: Poaceae (74.5%), Caryophyllaceae (9.8%), Solanacea (5.9%), Portulacaceae (3.9%), Lamiaceae (3.9%) and Brassicaceae (2.0%). We found that plants of the rice family (Poaceae) are important in the diet of wintering geese, especially for Greater white-fronted geese. This knowledge can be used to establish conservation strategies of the geese overwintering in South Korea.

## Introduction

Most species of geese migrate long distances between their breeding sites and wintering sites. Among them, the Bean goose (*Anser fabalis*) and Greater white-fronted goose (*Anser albifrons*) are the most common visitors in South Korea (Park & Won 1993). The breeding populations in the Asian arctic region is 140,000 for the Bean goose and 165,000–235,000 for the Greater white-fronted goose (Syroechkovskiy 2006). Among them, more than 50,000 Bean geese and 69,000 Greater white-fronted geese overwinter in South Korea (Ministry of Environment 2009, 2010, 2011, 2012, 2013).

For long-distance migratory birds such as the Bean goose and Greater white-fronted goose, it is important to build up their nutritional status before migrating back to their breeding grounds. In addition, the feeding status during the winter influences the productivity of the migratory birds in the subsequent breeding season (Robb et al. 2008). In spite of the recent attention to the conservational status of these geese (especially the Bean goose, which was designated as Endangered species level II in Korea; Rho et al. 2010), their ecology has rarely been studied in South Korea. In particular, understanding the diet composition is pivotal in establishing conservation strategies (Marrero et al. 2004; Valentini et al. 2009) but the diet composition of the geese during their wintering in South Korea has not yet been identified, except some descriptions on their consumption of ‘waste rice (Stafford et al. 2006)’ (Yoo et al. 2008).

Non-invasive samples such as feces, hair and feather are useful for species, sex and diet identification of endangered and/or elusive species (Sacchi et al. 2004; Horvath et al. 2005; Waits & Paetkau 2005; Deagle et al. 2007; Kim et al. 2011). For geese, most studies generally use invasive samples, such as blood and tissue samples (Quinn et al. 1991; Huang et al. 2003), and only a few studies used non-invasive samples, such as feathers (Kim et al. 2012; Kleven et al. 2016). Several non-invasive methods have been used for diet analysis of herbivores, including: (i) microscopical examination of plant cuticle fragments in fecal samples, (ii) chemical analysis of the natural alkanes of plant cuticular wax, (iii) near-infrared reflectance spectroscopy, and (iv) detection of plant DNA using PCR (Deagle et al. 2007; Valentini et al. 2009). The methods for plant DNA detection are still being developed (Oehm et al. 2011). However, diet analysis of the geese using non-invasive samples has not been conducted except one study on the Barnacle goose (*Branta leucopsis*) by Stech et al. (2011). Considering that geese are a representative group of migrating birds and their migration ecology is studied globally, the rarity of ecological studies using non-invasive samples is surprising. In this study, we conducted PCR-based analysis of the diet composition of Bean geese and Greater white-fronted geese using fecal samples collected in their wintering grounds in South Korea.

## Materials and methods

### Study area and sampling site

**Figure 1.**
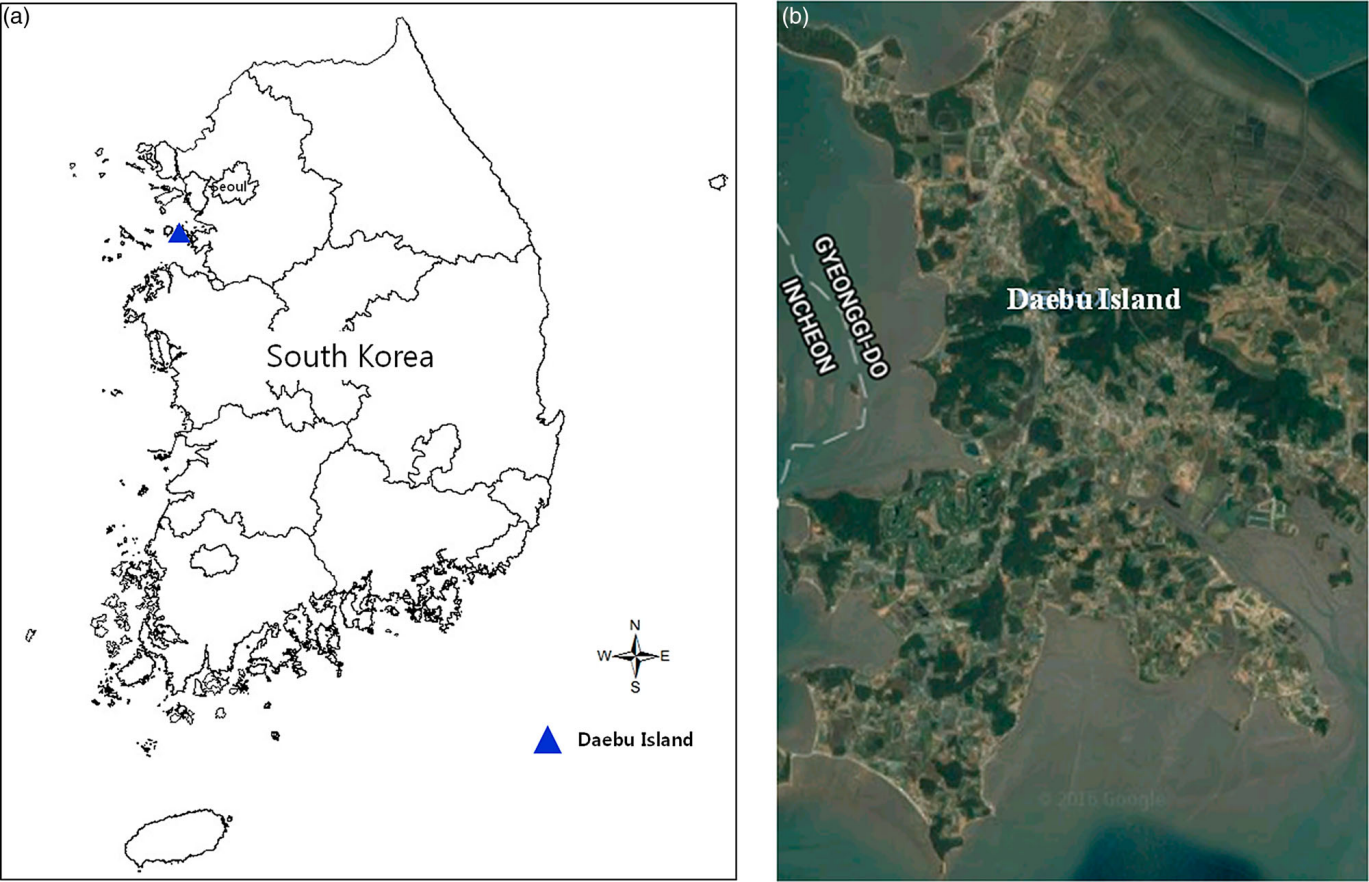
Location of the collection site. Location (a) and satellite map of Daebu Island (b).

The Bean goose and Greater white-fronted goose overwinter near the West and South coast region of Korea mostly in wetlands, such as reclaimed land or marsh (Kim et al. 2016). We selected Daebu Island (126°34′30″–126°39′0″E, 37°15′0″–37°16′30″N; Figure 1) on the West Coast as the collection site because it is one of the representative wintering sites of the geese in South Korea. Thirteen fecal samples of geese were collected in January 2010 in Daebu Island, Korea. Daebu Island includes a large reclaimed area that is recently serving as a main habitat for hundreds of bird species (Ministry of Environment 2004). Fecal samples of geese were frozen at −20°C right after the collection until used.

### Molecular methods

Genomic DNA was extracted from 13 fecal samples (up to 200 mg fecal samples) following Gerloff et al. (1995). Since the two geese species usually formed a mixed-species wintering flock, it was difficult to tell which fecal sample belonged to which species. Therefore, we first conducted species identification for the fecal samples using the method described by Kim et al. (2012).

For the diet analysis, we used a pair of primers, *rbc*L Z1aF and hp2R, to amplify the gene of large subunit of the ribulose-1,5-bisphosphate carboxylase (*rbc*L) from feces (following Kim et al. 2011). Genomic DNA was extracted from the fecal samples (100 mg out of the prepared fecal samples) following Gerloff et al. (1995). The chloroplast *rbc*L gene has been widely used to analyze the diet of herbivorous species (reviewed in Valentini et al. 2009). The PCRs were carried out in a 25 μl reaction volume containing 2 μl of DNA template, 2 mM of MgCl_2_, 1X PCR buffer (iNtRON Inc., South Korea), 0.2 mM of each dNTP, 0.1 μM of each primer, 2.5 μg of BSA (Promega Inc., USA) and 1U of *i-Star Taq* polymerase (iNtRON Inc., South Korea). The PCR amplifications were performed using PTC-100 PCR Thermal Cycler (MJ Research Inc., USA) with the following conditions: initial denaturation for 3 min at 94°C, followed by 50 cycles of amplification (94°C for 45 s, 55°C for 30 s and 72°C for 45 s) with a final extension for 3 min at 72°C. After purifying the PCR products on 3% agarose gel using Zymoclean^TM^ Gel DNA Recovery Kit (Zymo Research Corp., Japan), cloning was carried out with RBC T&A Cloning Kit under the manufacturer’s instructions (Real Biotech Corp., Taiwan). For colony sequencing, we conducted PCRs with universal M13F and M13R primers using the samples taken from colonies. After colony PCR, only the forward primer, M13F, was used for sequencing. All PCR products were directly sequenced using the ABI PRISM 3700 DNA sequencer (Applied Biosystems Inc., USA).

The length of the partial *rbc*L PCR product was 202 bp excluding the primer binding regions. The sequences were aligned with AlignIR program version 2.1 (LI-COR Inc., USA). These sequences were compared with *rbc*L sequences that are published in GenBank by means of nucleotide BLAST search, and the order and family of the closest matches were recorded. Statistical comparison of the two geese species in their diet composition (at the family level) was conducted with chi-square tests.

In order to provide more detailed understanding of the diet composition, we presented the list of genera showing highest Max scores and identity values recognized by BLAST search. We additionally provided the identity of the plant species by comparing the list of genera recorded from BLAST search with the species list of vascular plants surveyed in Daebu Island (Lim et al. 2014).

## Results and discussion

Among 13 fecal samples, four were identified to be from the Bean goose. From the 4 samples, we obtained a total of 36 clones containing plant *rbc*L genes. From the 36 clones, a total of 4 orders and 5 families of plants were recognized (Table 1). Caryophyllaceae (75.0%) was the most dominant diet plant, followed by Poaceae (13.9%), Asteraceae (5.5%), Polygonaceae (2.8%) and Cucurbitacea (2.8%) (Table 1).

**Table 1.** Plants in the diets of the bean goose and greater white-fronted goose in Daebu Island.

Order	Family	Bean goose		Greater white-fronted goose	
		No. of clones	% of clones	No. of clones	% of clones
Poales	Poaceae	5^a^	13.9	38^a^	74.5
Caryophyllales	Caryophyllaceae	27	75.0	5	9.8
	Polygonaceae	1	2.8		
	Portulacaceae			2	3.9
Brassicales	Brassicaceae			1	2.0
Solanales	Solanacea			3	5.9
Lamiales	Lamiaceae			2	3.9
Cucurbitales	Cucurbitacea	1	2.8		
Asterales	Asteraceae	2^a^	5.5		
Total		36	100	51	100

^a^Sequences from one clone could also be assigned to a genus that does not belong to this taxonomic group. Refer to Table 2 for the details.

The rest of fecal samples (*n* = 9) were identified to be from the Great white-fronted goose. From these samples, a total of 51 clones with plant *rbc*L genes were successfully sequenced and a total of 5 orders and 6 families of plants were identified (Table 1). Poaceae (74.5%) was the most dominant diet plant, followed by Caryophyllaceae (9.8%), Solanacea (5.9%), Portulacaceae (3.9%), Lamiaceae (3.9%) and Brassicaceae (2.0%) (Table 1). The diet composition estimated in this study at the family level was different between the two geese species (*χ*^2^*^ ^*= 139.98, *P* < .001).

Many genera that recorded the highest scores from the BLAST search were also found in the catalogue of the vascular plants previously surveyed in the study site (Table 2). In addition, two genera for crops (such as rice *Oryza* or daikon *Raphanus*) were identified from the list of genera obtained from BLAST search. This suggests that our molecular method can be used as a valid method for estimation of the diet composition of the geese from non-invasive samples.

**Table 2.** Plants from the feces of the Greater white-fronted geese and Bean geese. The genera that were present in the catalogue of vascular plants surveyed at the study site (Lim et al. 2014) are marked with bold; the genera that were likely to be present but not listed in the catalogue (such as crops) are underlined.

	Order	Family	List of genera with the highest score from BLAST search
Bean goose	Poales	Poaceae	*Aira, **Arundinella**, Castellia, Chikusichloa, Coelachne, Deschampsia, **Festuca***, *Helictochloa, Helictotrichon, Lamarckia, **Lolium**, Oryza, **Phragmites**, Rhynchoryza, Sesleria, Vulpia*
	Caryophyllales	Caryophyllaceae	***Cerastium****, Myosoton,****Silene****, **Spergularia**, **Stellaria**, Viscaria*
		Polygonaceae	*Bistorta,****Persicaria****, **Polygonum***
	Cucurbitales	Cucurbitaceae	***Trichosanthes***
	Asterales	Asteraceae	***Aster****, **Youngia***
	Alismatales	Hydrocharitaceae	***Hydrilla*** (recognized with Poaceae from the same clone)
	Laurales	Lauraceae	*Persea* (recognized with Asteraceae from the same clone)
Greater white-fronted goose	Poales	Poaceae	*Ancistrachne, Arthrostylidium, **Arundinella**, Aulonemia, Bambusa, Borinda, Buergersiochloa, Chikusichloa, Coelachne, Dendrocalamus, Dichanthelium, **Echinochloa**, Elytrophorus, **Eragrostis**, **Eriochloa**, Greslania, **Leersia**, Loudetia, Melocanna, Neololeba, Oryza,****Panicum****, Phalaris, **Phragmites**, Rhipidocladum, Rhynchoryza, Schizostachyum, Scutachne, Thyrsostachys, Yakirra, **Zizania***
	Caryophyllales	Caryophyllaceae	***Cerastium****, Myosoton, **Silene**,****Spergularia****,****Stellaria***
		Portulacaceae	***Portulaca***
	Brassicales	Brassicaceae	*Raphanus*
	Solanales	Solanacea	*Brugmansia, Capsicum, **Datura**, Dunalia, Iochroma, Saracha, **Solanum**, Vassobia, Withania*
	Lamiales	Lamiaceae	***Isodon***
	Alismatales	Hydrocharitaceae	***Hydrilla*** (recognized with Poaceae from the same clone)

A drawback of using BLAST search for estimating the diet composition is that some ambiguities can arise. In our analyses, there were two ambiguous cases: The *rbc*L sequence from one clone showed the highest matching scores (100%) with two genera (*Phragmites* and *Coelachne*) from Poaceae and *Hydrilla* from Hydrocharitaceae. Plant species belonging to these three genera were found in the vascular plant list reported in Daebu Island. We could frequently observe that both geese species feed the roots of plants belonging to *Phragmite* and *Coelachne*. In contrast, we have not observed that the geese forage plants belonging to *Hydrilla* (Kim BJ, Pers. Obs)*.* Based on this observation, we think the likelihood that *Hydrilla* from Hydrocharitaceae is present in the diet of the geese is low. Similarly, *Persea* from Lauraceae was recognized together with a genera (*Youngia*) from Asteraceae for the *rbc*L sequence from the other clone with 98% identity values. In the vascular plant list recorded from the study site, one plant species belonging to *Youngia* is present but no species from *Persea* is present. Thus, we think that it is plausible that *Persea* is not present in the diet of the geese. Such ambiguities can be circumvented by amplifying longer sequences and/or using less conservative genetic marker than *rbc*L in future studies.

Our results show that, even though the Bean goose and Greater white-fronted goose form mixed-species wintering flocks and co-occur in many wintering grounds in South Korea, the diet composition of the two species differs. Although their ecological niches are often assumed to be identical, detailed use of habitats between the two species differed (Kim et al. 2016). Presumably, Greater white-fronted geese prefer rice paddies, whereas Bean geese use wetlands. As we used a small number of fecal samples and clones in the present study, a larger-scale study has to be conducted in order to fully ascertain the difference of diet composition of these two geese species.

In winter, the Bean goose and Greater white-fronted goose mostly feed on crops, such as rice left on the rice fields (Pers. Obs; Yoo et al. 2008). In our results, the main component of the diet of the Greater white-fronted goose was Poaceae which includes rice. For the Bean goose, the family Poaceae constituted the second major item in their diet. Considering that we had small samples for the Bean goose and the main diet of a related species, Lesser white-fronted goose (*A. erythropus*) wintering in the Evros Delta, Greece, was rice (Karmiris et al. 2014), it is plausible that many geese species heavily rely on the waste rice in wintering grounds. The importance of rice in conservation of diverse bird species, especially waterbirds, is already recognized by many studies (e.g. Stafford et al. 2006; Stafford et al. 2010). Populations of the Greater white-fronted goose wintering in Korea have increased in mid-2000s (Syroechkovskiy 2006), and this may be related to the increase in agricultural lands due to the reclamation project on the West Coast of Korea (Kim et al. 2016).

Even though our study was a preliminary one involving small number of samples, it shows the usefulness of non-invasive samples for diet analysis of herbivores and promotes further molecular studies of similar scopes. Our preliminary results can also be used in collecting information necessary for establishment of conservation strategies for geese populations overwintering in East Asia where the rice fields are abundant. In the future, a new advanced technique such as Next Generation Sequencing (NGS) of plant DNAs from fecal samples, would be applicable for the diet analysis as an alternative of cloning technique.
